# Recent Advances in Electrospun Sustainable Composites for Biomedical, Environmental, Energy, and Packaging Applications

**DOI:** 10.3390/ijms21114019

**Published:** 2020-06-04

**Authors:** Hao Liu, Christopher R. Gough, Qianqian Deng, Zhenggui Gu, Fang Wang, Xiao Hu

**Affiliations:** 1 Center of Analysis and Testing, Nanjing Normal University, Nanjing 210023, China; 181135012@stu.njnu.edu.cn (H.L.); 191135031@stu.njnu.edu.cn (Q.D.); 2 School of Chemistry and Materials Science, Nanjing Normal University, Nanjing 210023, China; 07160@njnu.edu.cn; 3 Department of Physics and Astronomy, Rowan University, Glassboro, NJ 08028, USA; goughc2@students.rowan.edu; 4 Department of Chemistry and Biochemistry, Rowan University, Glassboro, NJ 08028, USA; 5 Department of Biomedical Engineering, Rowan University, Glassboro, NJ 08028, USA; 6 Department of Molecular and Cellular Biosciences, Rowan University, Glassboro, NJ 08028, USA

**Keywords:** electrospinning nanofiber, biomedical, environmental, energy, food packaging material

## Abstract

Electrospinning has gained constant enthusiasm and wide interest as a novel sustainable material processing technique due to its ease of operation and wide adaptability for fabricating eco-friendly fibers on a nanoscale. In addition, the device working parameters, spinning solution properties, and the environmental factors can have a significant effect on the fibers’ morphology during electrospinning. This review summarizes the newly developed principles and influence factors for electrospinning technology in the past five years, including these factors’ interactions with the electrospinning mechanism as well as its most recent applications of electrospun natural or sustainable composite materials in biology, environmental protection, energy, and food packaging materials.

## 1. Introduction

**Figure 1:**
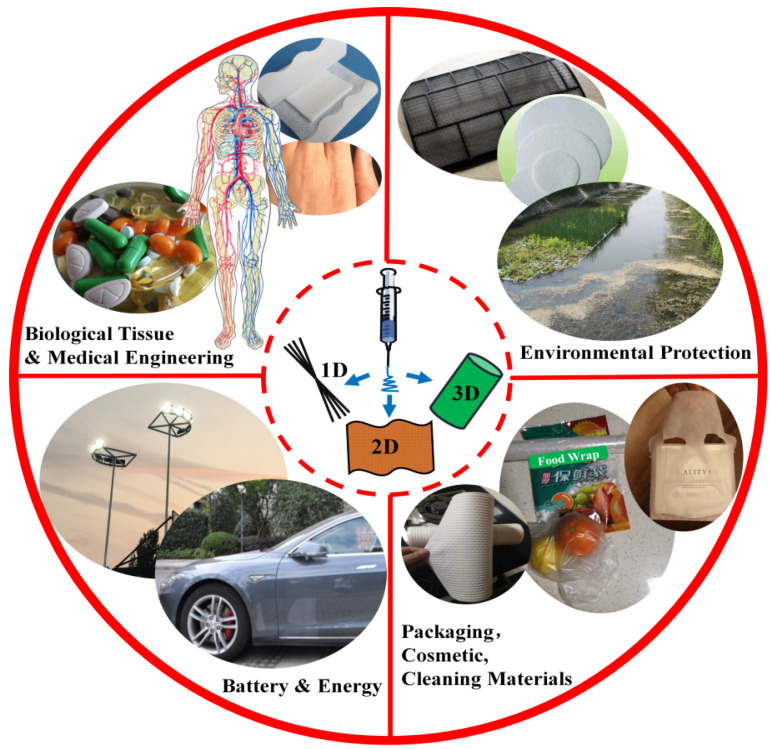
The electrospun nanofibers that can be assembled to 1D, 2D, and 3D structures, and their related sustainable applications. These nanofibrous materials are of special properties, such as superhydrophobicity, superhydrophilicity, superconductivity, and adjustable mechanical properties, which have led to widespread ecofriendly applications in batteries, environmental consciousness, biological tissue, and medical engineering, as well as food packaging and cosmetic materials.

Materials made from electrospinning technology (EST) have been created for use in a variety of applications in several fields due to their small diameter, large specific surface, and high porosity [1,2,3,4,5]. For instance, nanofibers created from electrospinning technology can be used for biological engineering, pollution treatment, and as sustainable energy materials [6,7,8,9,10,11,12,13]. Electrospinning technology refers to a method in which a polymer solution or a melt is spray-stretched and then volatilized or melt-cured in order to create an ultrafine fiber using a high-voltage electrostatic force. Since Rayleigh et al. discovered the technique of electrospinning that results from a liquid jet under electrostatic forces one hundred years ago, the technique has continuously attracted much attention, such as in the preparation of nontissue membranes, acrylic fibers, and various nanofibers [14,15,16]. Biologically, electrospinning has been used for wound dressings and for sustained-release drug materials to maintain a stable release of drugs and avoid the harmful effects of overdosing [17,18,19,20,21,22,23,24,25,26]. Simultaneously, electrospun materials have also been applied to the field of tissue engineering to replace damaged tissue and repair the function of native tissue, including soft bone, vascular, and nerve tissues [27,28,29,30,31,32]. Meanwhile, the rapid development of nanotechnology has also provided new sustainable applications for electrospun materials [33,34,35,36,37,38,39,40,41]. For example, electrospun nanomaterials could be used to adsorb pollutants in air and water due to their large specific surface area and high porosity [42]. Therefore, the electrospinning technique could supply a new strategy for producing ecofriendly nanomaterials with special properties (Figure 1).

This review will begin with a brief introduction of the principles behind electrospinning principles, and then go on to explain the preparation of electrospun materials and the effects of various factors on the properties of these materials. Afterwards, a detailed review of the recent (mainly 2016–2020) applications for electrospun sustainable composite materials in biological engineering, environmental protection, and within the energy and food packaging industry, will be given.

## 2. Electrospinning Technology

Generally, an electrospinning technology allows the polymer solution to pass through an electric field established between the nozzle and the receiver to form nanofibers. Under the electric field force, the number of charged particles in the droplet on the nozzle increases, which leads the outermost droplet of the nozzle to be stretched into a cone shape and moved towards the receiver when the electric field force on the droplet surface is greater than the surface tension. During the ejection and movement of the polymer droplets, the solvent in the droplets quickly evaporates and solidifies, and finally, the nanofibers are collected on the receiving device [18,19,43].

### 2.1. Electrospinning Devices

**Figure 2:**
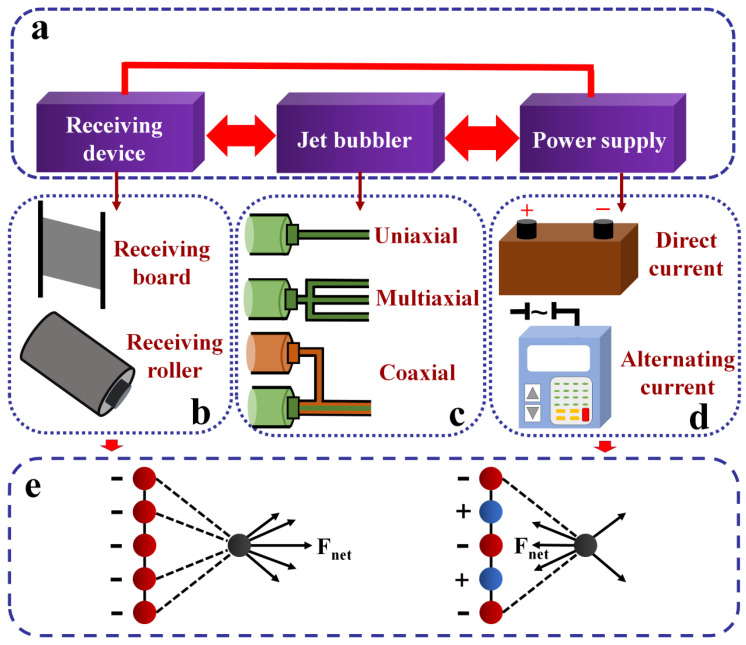
(**a**) Schematic diagram of an electrospinning device for sustainable materials, which consists of three parts: (**b**) a receiving device, often a flat metal plate or a rotating drum, (**c**) a jet bubbler which mainly includes uniaxial, multiaxial or coaxial configurations, and (**d**) a power supply, including direct current (DC) or alternating current (AC). (**e**) Electrostatic mechanism diagram shows the forces acting on a displaced segment of an electrically charged jet in DC electrospinning (left) and AC electrospinning (right) [40,57,59,61]. Figure 2e is reproduced with permission from [55]; copyright (2007) Wiley.

An electrospinning device is mainly composed of three parts: receiving device, jet bubbler, and power supply (Figure 2a). Different types of sustainable materials can be produced by changing the components of the electrospinning equipment. For example, some fibers can be spun onto a plate, while others could be spun under a rotating drum (Figure 2b). Moreover, single-strand fiber can be spun with a single-axis needle, while multiaxis needles can be used to spin multicomponent fibers at the same time, and multilayer wrapped fiber can be spun with coaxial spinning (Figure 2c). Both alternating current (AC) and direct current (DC) can be applied from the electrical power supply of the electrospinning setup (Figure 2d). The electrostatic mechanism that drives electrospinning in DC and AC electrospinning setups is illustrated in Figure 2e.

#### 2.1.1. Direct Current (DC) Electrospinning

The basic DC electrospinning device consists of a high voltage DC power supply, a shower head, and a receiving device. The receiving device is typically a flat metal plate or a rotating roller (Figure 2b), which can produce either randomly oriented nonwoven mats or aligned nanofibers, depending on the fiber collection methods used [44]. The high voltage end, usually positive, is directly connected to the spinneret during the electrospinning process, while the receiving device is linked to a negative terminal or ground [45]. The electric field is generated between the syringe needle and the receiving device when a high voltage is supplied [45,46]. Meanwhile, surface tension maintained on the fluid droplet will be subjected to two types of electrostatic forces at the tip of the spinneret, which are the mutual repulsive force between the same charge on the droplet surface and the Coulomb force caused by the external electric field [45,46]. The suspended charged droplet at the metal needle gradually forms a Taylor cone with increasing voltage. If the voltage continues to increase, the tip of the Taylor cone will overcome the surface tension to generate a solution jet [46,47]. A nanofiber is fabricated when the viscoelastic jet derived from a polymer solution is continuously stretched by the electrostatic repulsions and the evaporation of the solvent [48,49]. According to a recent study [50], a flexible and cytocompatible poly(glycerol sebacate) and poly-L-lactic acid (PGS/PLLA) fibrous scaffold with a core–shell structure was fabricated by coaxial DC electrospinning under 20 kV voltage, which could be used to replace natural tissues for speeding up skin regeneration [50]. Yu et al. [51] have also reported that 1D carbon matrix composite nanofibers were fabricated using a simple DC electrospinning method under 12 kV applied voltage. Liu et al. [52] reported a novel 3D biomimetic-coated composite scaffold produced with polycaprolactone (PCL) and polylactic acid (PLA) solutions (solvents are dichloromethane (DCM) and *N*,*N*-dimethylformamide (DMF)) by combining electrohydrodynamic (EHD) jetting, and DC electrospinning and coating techniques. The scaffolds have good potential for skin tissue engineering and wound healing. The traditional DC electrospinning technique has been used as an efficient processing method for the fabrication of various types of nanofibers with different nanocompositions and structures, which have been used for a broad range of applications, such as filtration membranes, catalysts, electronic devices, and biomedical scaffolds [53]. However, DC power can give the polymer solution droplet a charge of a single polarity (Figure 2e, left), which primarily leads to fiber deposition, an undesired side-effect [54]. Therefore, electrospun fibers from a DC power source are electrically charged and inherently unstable during formation, which usually causes the mats produced from the fibers to exhibit a random, nonwoven microstructure [55]. Using a DC power source also has some deficiencies in its application, such as low production efficiency, poor stability, and difficulty in collecting the fibers [56].

#### 2.1.2. Alternating Current (AC) Electrospinning

There is not much difference in composition between the AC and DC electrostatic spinning devices, except the AC experiment setup is equipped with a voltage transformer fed by a 0~230 V variable transformer (Figure 2d). The sinusoidal alternating power source voltage is controlled by a variable transformer. Through this setup, charge accumulation on the fibers is minimized due to the positive and negative charges neutralizing each other in the alternating regions of fiber when AC voltage is applied. This results in increased stabilization and alignment of the resultant fibers as charge repulsion effects are reduced compared to DC electrospinning [55,57,58]. As shown in Figure 2e (right), the forces are balanced on a displaced segment of an electrically charged jet. Many researchers [57,58,59,60,61,62,63] have demonstrated the possibility of increasing the alignment of electrospun fibers by using an AC voltage instead of a traditional DC voltage. Tepper and co-workers showed that nanofibers of PEO collected on the rotating mandrel had greater alignment when using an AC rather than DC power supply [57,59]. In another study [60], beadless PCL fibers with diameters tunable from 150 to 2000 nm could be rapidly manufactured under the range of 15–38 kV rms AC voltage. Likewise, Stanishevsky et al. [61] published a study using two similar alternating current systems capable of producing AC-voltages up to 40 kV rms, operated either at 60 or 50 Hz, respectively, to create a precursor to nanofibers. These nanofibers showed ample promise in gas filtration, separation, and other applications. Researchers have also been able to obtain nanofibrous layers made from a 20% concentration of gelatin in a solvent system containing acetic acid, distilled water, and ethanol at a voltage of 34 kV and a frequency of 50 Hz [62]. Compared with DC high voltage spinning, the newly developed AC electrospinning technology can decrease the charge accumulation on the fiber, reduce the charge repulsion effect, and improve the stability of the fiber [62]. Therefore, it was found that the fibers produced from AC electrospinning were more stable, more efficient, easier to refine, and easier to collect [45,56,63].

### 2.2. Factors Influencing the Electrospinning Process

There are three main factors that can affect the electrospinning of fibers: the electrospinning fluids, the operating conditions, and the environmental factors [4,64,65,66,67,68,69]. Table 1 summarized the different influence factors and their effects on fiber morphology.

#### 2.2.1. The Effect of Electrospinning Fluid Properties

The properties of electrospinning fluids include liquid viscosity, electrical conductivity, surface tension, and so on. Viscosity is a macroscopic reflection of the solute concentration in the electrospinning liquid. When the viscosity is too low, continuous fibers will not be produced. As the viscosity increases, the spinning-like shape changes from granular to spindle-shaped, and the fibers gradually become uniform and their diameter gradually increases. However, when the viscosity exceeds a certain range, the solution may become too viscous to pass through the nozzle or the resulting fibers may not be uniform and may stick together [64,65,70]. Uyar et al. [66] investigated the influence of different solvents during electrospinning and showed that spinning solutions with high conductivity could be spun into uniform and fine fibers, while beaded fibers might be obtained under low conductivity. Wang et al. [71] proposed that increasing the electrical conductivity of the electrospinning liquid can promote the stretching of the jet in the electric field, thereby making the fiber diameter smaller. Therefore, it is believed that the conductivity can affect the morphology of the fiber. During the electrospinning, a jet of fibers can be formed only when the electric field force at the nozzle, subjected to the droplet surface, is greater than the surface tension of the solution [67]. Increasing surface tension can reduce the surface area and make the jet change to a spherical shape. Together, a high voltage and a high solution viscosity can inhibit the jet shape from changing rapidly and facilitate the smooth and uniform formation of fibers [67]. The solvents used in electrospinning can also play a vital role in biological applications. Different solvents, such as dichloromethane (DCM), *N*,*N*-dimethylformamide (DMF), 2,2,2-trifluoroethanol (TFE), as well as 1,1,1,3,3,3-hexafluoro-2-propanol (HFP), have been extensively used to prepare spinning solutions, especially for dissolving biopolymers [68]. Bazrafshan et al. [68] summarized the spinnability of purified collagen with excellent biological properties. They elaborated that the polarity of the solvent may lead to the biodegradation of the triple-helical conformation of polycationic biopolymers (such as collagen), while higher polymer concentrations are able to generate more uniform fibers. Chan et al. [4] used hexafluoroisopropanol or water as a solvent to prepare aortic vascular grafts from natural silk fibroin. They found that hexafluoroisopropanol promotes better endothelialization and a more stable formation of new inner membranes compared to water.

#### 2.2.2. The Effect of Operating Conditions

The operating conditions of electrospinning include the voltage, needle size, receiving distance, and spinning solution flow rate. Among them, the voltage is a decisive factor in the formation of the electrospun fiber, since when the applied voltage exceeds the critical voltage, a stable jet can be ejected. However, if the voltage exceeds a certain range, the motion time of the jet in the electric field is reduced, so that the solvent cannot be completely evaporated, thereby affecting the uniformity of the fiber [72]. The receiving distance between the nozzle and the gathering device can affect the electric field strength, the flight time, and the stretching distance of the jet in the electric field. For a certain voltage, a short receiving distance can lead to too strong of an electric field and too short of a stretching distance, resulting in difficulty volatilizing the solvent and fiber nonuniformity, while a long acceptance distance with ample stretching time and length allows the solvent to volatilize sufficiently, creating uniform small-diameter fibers [69]. For beaded fibers, as the receiving distance increases, the bead is stretched and increased. It has been reported that reducing the flow rate will also reduce the diameter of the fibers and beads [73]. Megelski et al. [74] pointed out that a higher flow rate might result in thicker fibers. Akhgari et al. [75] also believed that the velocity of the electrospinning fluid had a significant effect on the fiber diameter. The size of the needle also has a certain effect on the fiber diameter. In general, a decrease in needle size leads to a decrease in the fiber diameter. Katti et al. [76] investigated the effect of needle size on nanofibers prepared from biodegradable polymers. The results show that the average diameter of the nanofibers decreases with a decrease in the orifice diameter of the needle.

#### 2.2.3. The Effect of Environment Conditions

The temperature and the humidity during experiments are the main environmental factors affecting nanofiber formation. Increasing the temperature can promote the evaporation and curing of the solvent while reducing the viscosity of the electrospinning fluid. However, excessively high temperatures will cause the jet to solidify in advance in the electric field, destroying the uniformity of the fiber [77]. Similarly, the humidity can affect the solvent evaporation greatly. Low humidity can promote the solvent’s evaporation, while high humidity makes evaporation of the solvent difficult. Therefore, specific nanofiber morphology can be acquired by adjusting the environmental humidity [78]. On the other hand, low humidity can increase the charge density on the electrodes and cause air breakdown. With the increase of relative humidity, due to the increase of water vapor molecules in the air, the charge carried by the jet is easily transferred to the environment, thereby reducing the surface charge density of the jet and enabling the electrostatic spinning to proceed normally [79].

In summary, the most significant factors influencing fiber formation during electrospinning are the operating conditions, spinning solution properties, and environmental factors. They are crucial for the stability of the electrospinning process, fiber diameter and uniformity, and fiber morphology, which lays a theoretical foundation for better use of electrospinning technology to prepare new nanomaterials suitable for different fields.

**Table 1:** Three main factors affecting electrospun sustainable fiber materials.

Factor	Parameter	Point of Action	Reference
Properties of electrospinning fluid	Liquid viscosity	Fiber diameter and uniformity	[2,28,64,68,70,74,80,81,82,83,84,85,86,87]
	Electrical conductivity	Fiber diameter and distribution	[2,28,66,70,71,81,84,87,88,89,90]
	Surface tension	Fiber formation	[64,67,68,70,91,92]
Operating conditions	Voltage	Fiber diameter	[68,72,76,83]
	Needle size	Fiber diameter	[65,69,76]
	Receiving distance	Solvent volatilization and fiber diameter	[69,74]
	Spinning solution flow rate	Fiber diameter	[74,75,93]
Environment conditions	Temperature	Solvent volatilization and liquid viscosity	[28,77]
	Humidity	Solvent volatilization	[78,79]

## 3. Application of Electrospun Sustainable Composite Materials

Compared to conventional methods, such as melt-spinning and wet-spinning, electrospinning is a simple and practical method for preparing different functional nano- or microfibers with high specific surface areas and good homogeneity at a low cost for a wide variety of applications [94,95]. Many types of sustainable materials have been fabricated by electrospinning, including adsorption [96] and filter materials [71] used in environmental management, bioengineering materials with biological affinity [28], and drug delivery vehicles that regulate the rate of drug release [97]. For example, as a manufacturing technique for biomedical materials, Meng et al. [98] electrospun gelatin/polyvinyl alcohol-based materials, and Cheng et al. [28] electrospun a biodegradable cellulose nanocrystal membrane. Both of them overcome the limitation of cell adhesion and migration by adjusting the hydrophobicity of the nanofiber membrane. Table 2 lists the recent applications of electrospinning different polymeric materials in the fields of biology, environmental protection, energy, and packaging materials. Their solvents and operating parameters are summarized in detail with the literature. It shows that the selection of working parameters (such as electrospinning voltage, receiving distance, and solution flow rate) is crucial for various materials and solvents during the spinning process.

### 3.1. Recent Biological and Medical Engineering Applications

As mentioned above, the electrospinning technology has been regarded as one of the most effective methods for preparing wound dressings and nanoscale drug carriers/delivery due to its cost-effectiveness and ease of operation [45,56]. A drug delivery system with controllable release helps avoid overdosing problems, minimizing the chances of a drug being toxic to patients. Tissue engineering materials require further properties, including sufficient permeability to allow cells embedded on the material to exchange oxygen and carbon dioxide with the environment, in order to minimize the risk of infection [99]. Ferulic acid (FA), one of the main biological phenolic acids, has many desirable biological properties, such as antioxidative, antibacterial, and antitumoral properties. However, it is hydrophobically unstable and is metabolized quickly by the body, giving it a short window of availability in biological applications. Yakub et al. [80] used dimethylformamide and tetrahydrofuran as a solvent to electrospin polycaprolactone (PCL) and chitosan embedded with FA, and found that the nanofiber with FA possessed good antioxidant activity, killed staphylococcus aureus, and exhibited significant antitumor properties over fibers lacking FA. Zhang et al. [100] electrospun a collagen/polyvinyl alcohol drug delivery system coated with salicylic acid. They illustrated that the porous nanofiber microspheres could reduce the drug release rate, protect its morphology in solution, and control drug release effectively. Akhgari et al. [75] electrospun polyvinylpyrrolidone (PVP) nanofibers containing chloratadine and found that the best parameters for uniform nanofibers with a quick drug release were 30% concentration of PVP in ethanol, 1:4 ratio of drug to polymer, 10 kV working voltage, and a 1 mL/h rate. In addition, some research [81,88,101] has shown that electrospinning could produce stimulus-responsive fibers. For example, the difference between pH at normal physiological conditions and pH in the tumor microenvironment has promoted the development of pH-sensitive drug delivery systems generated by electrospinning technology [81]. Sang et al. [88] have reported that poly(lactide-co-caprolactone) (PLCL) fibers and hydrophilic gelatin fibers can both be doped with sodium bicarbonate (SB) to increase their pH sensitivities. Hydrophilic gelatin fibers released ciprofloxacin faster than their hydrophobic PLCL analogs. Gelatin/SB fibers were more sensitive to pH compared to fibers without SB. The researchers suggested that their gelatin/SB fiber was suitable as an implantable agent for the prevention of wound infection in vivo after tumor resection [88].

**Figure 3:**
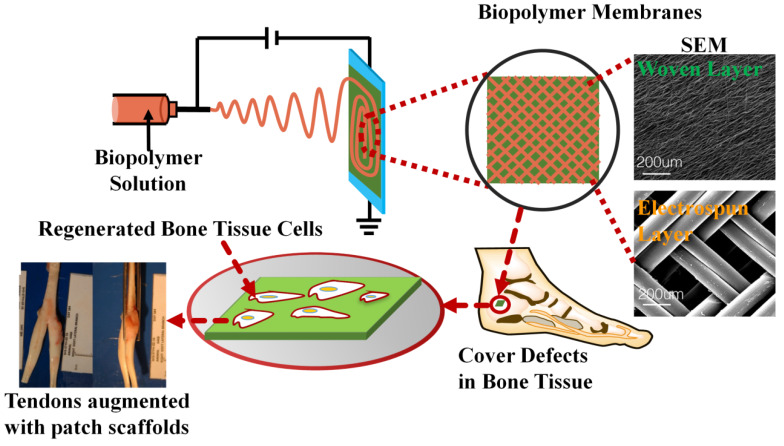
Blended electrospun biopolymer materials can be used to cover the damaged bone tissue and to promote the growth of bone cells [107]. Reproduced from [107]; Open access copyright (2020) Springer Nature.

Simultaneously, tissue engineering applies the principles of material engineering and the life sciences to manufacture functional tissue material to replace diseased or damaged organs and restore the functionality of these organs without having to subsequently remove them [101,102,103,104,105,106,107,108]. Electrospinning can be employed to create porous three-dimensional structures to which cells can adhere, proliferate, grow, and differentiate in order to repair and replace damaged tissue [109,110,111]. Kim et al. [7] demonstrated that the morphological factors of electrospun fibers have a significant effect on the growth mode of neuronal cells by showing that neurites grow along the direction of the fibers, subsequently decreasing the linearity of the neurites when fiber density increases. Wang et al. [12] fabricated three-dimensional polymer scaffolds with mechanical properties similar to tendons with controllable anisotropic microstructures, which could be used to deliver drugs or for tissue regeneration. Roy et al. [112] chose biodegradable polycaprolactone (PCL) and natural silk fibroin (SF) polymer for electrospinning. The experimental results showed that the nanofibers had better cell biocompatibility and could promote cell growth. Interestingly, filling damaged bone tissue with electrospun nanofiber materials made of biopolymer materials can not only promote the growth of bone cells, but also accelerate the formation of new bone at the damaged area (Figure 3). Lotfi et al. [113] evaluated the biocompatibility and osteogenic differentiation of mesenchymal stem cells (MSCs) in vitro acting on electrospun nanofiber membranes. It was confirmed by a quantitative real-time polymerase chain reaction that the electrospun chitosan-coated collagen nanofiber membrane induced osteogenic genes in MSCs and guided bone regeneration, showing the potential for this system to accelerate the induction of new bone formation. Similarly, Yin et al. [114] used a coaxial electrospinning technique to fabricate silk fibroin and poly(l-lactide-co-caprolactone) 3D nanofiber membranes and evaluated the physicochemical and biological properties of these novel membranes by using scanning/transmission electron microscopy, laser confocal microscopy, and X-ray diffraction analysis. They found that the diameter and shape of this electrospun fiber film were the best when the voltage was 15 kV, the working distance was 15 cm, and the flow ratio of the shell/core was 1:10. This bifactor-loaded coaxial electrospun fiber membrane could effectively improve cell viability and early osteogenesis in bone defect repairment. Oyama et al. [115] obtained a poly-l-lactic/acid-polycaprolactone (PLLA/PLCL) electrospun membrane by using electrospinning technology as a scaffold for cell growth. The feasibility of electrospinning scaffolds for biomaterials was verified by mechanical experiments and electron microscopy observation. By comparing the mass ratio of PLLA/PLCL at 10:90, 30:70, and 50:50, the melting temperature of the mixture increased with the increase of PLLA concentration, which might be due to the separation and crystallization induced by higher PLLA concentration, growing more stable crystals. They found that after 72 h, the number of living cells was 5 times higher than that at the beginning of the experiment, which proved the feasibility of using the spinning mixture film as the matrix to culture adherent cells. Khan et al. [116] prepared dual-network electrospun tubes from poly(1,4-cyclohexanedimethylene isosorbide terephthalate)/ poly(vinyl alcohol) (PVA) hydrogel, which was capable of meeting the requirements of toughness and size in physiology. Shao et al. [117] simulated the scaffold of extracellular matrix structure in natural bone by embedding hydroxyapatite-tissue silk fiber nanoparticles into the homogenized silk nanofiber using coaxial electrospinning technology. Characterization results showed that the mechanical properties of the composites were significantly improved compared to the pure tussah silk, and could support cell adhesion and proliferation, and promote the deposition and biomineralization of alkaline phosphatase and minerals. The investigators believed that this kind of composite represents a new biomaterial that can be used as a bioscaffold in tissue and bone regeneration.

### 3.2. Recent Environmental Engineering Applications

**Figure 4:**
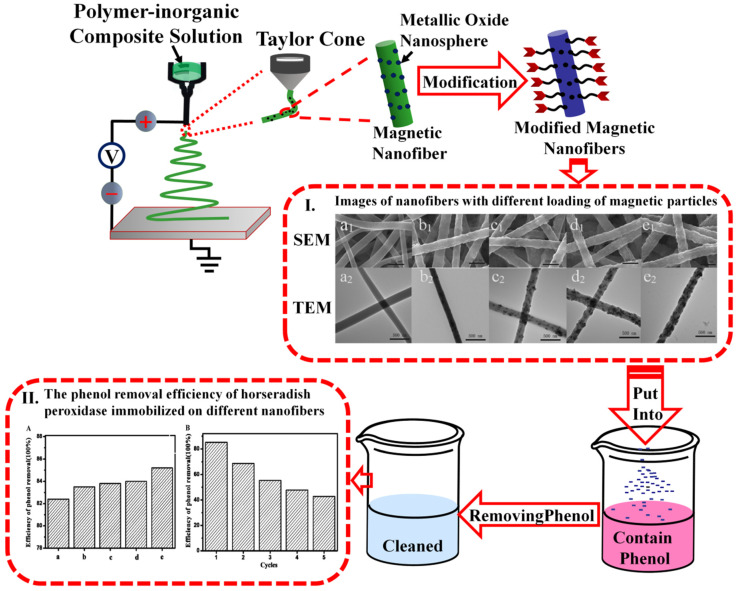
Nanorods prepared from polymer inorganic composite solutions containing metal nanoparticles can be used to treat phenol and other pollutants in water after modification and other processes. Reproduced with permission from [125]; copyright (2018) Elsevier.

Electrospun materials can also be used in filters and for the adsorption of pollutants from the environment. For example, nanomaterials prepared from polymer inorganic composite solutions containing metal nanoparticles can be used to treat phenols and other pollutants after they have been modified, and have great potential for applications in the field of environmental governance (Figure 4) [118,119,120,121,122,123,124]. Li et al. [125] prepared Fe_3_O_4_/polyacrylonitrile (PAN) nanofibers by adding Fe_3_O_4_ magnetic nanoparticles to a PAN electrospinning solution and treated the Fe_3_O_4_ magnetic nanoparticles on the surface of the nanofibers with dopamine and glutaraldehyde. Horseradish peroxidase (HRP) was successfully fixed to the magnetic nanoparticles on the surface of the fibers. The obtained nanofibers were added to a phenol solution containing H_2_O_2_. The results illustrated that a minimum 40% loading of Fe_3_O_4_ nanoparticles in HRP possessed the highest relative activity due to the magnetic synergy of Fe_3_O_4_. The catalytic phenol removal efficiency at the first use was 85%, and the efficiency was still 52% after five consecutive uses. Dai et al. [119] prepared multiwalled carbon nanotube modified laccase-carrying electrospun fibrous membranes (MWCNT-LCEFMs) by combining electrostatic spinning nanofiber technology with multiwalled carbon nanotubes which could effectively remove phenolic contaminants. After two months, the laccase in MWCNTs-LCEFMs still retained more than 82% of the initial activity, and the laccase activity recovered to above 92% after coating in 2,2-azinobis-3ethylbenzothiazoline-6-sulfonate, which showed the best removal effect on phenolic pollutants. They also found that the storage laccase performance of fiber materials was enhanced significantly as the content of MWCNTs reached 1.5 wt%, with the highest efficiency for removing phenolic pollutants.

In addition, the large amount of gas containing different pollution particles emitted from factories may not be treated effectively, while fine particles could also cause serious damage to the lungs and health of humans [72,126,127,128]. Al-Attabi et al. [82] explored the aperture and distribution for spinning fiber, which could filter tiny particles in the air. When the mass concentration of the polymer was 12%, the obtained nanofiber membrane had the best filtration effect for particles with a diameter of 300–500 nm. Li et al. [83] used N,N-dimethylformamide (DMF) to dissolve polyimide and electrospun polyimide nanofibers on aramid nonwoven substrate through high-temperature adhesives. They found that this sandwich structure material could completely filter particles with a diameter greater than or equal to 2.0 μm, and the removal rate was up to 99.5%. Yan et al. [79] electrospun an ultrafine polyamide-6 nanofiber membrane that could filter 300–500 nm NaCl particles at an 85 L/min rate and reduce the concentration of PM2.5 from 999 to 34.1 μg/m^3^ in 10 min. Simultaneously, its filtration efficiency was as high as 99.42%. Yu et al. [129] reported the formation of multilayer nylon-6 nanofibers by electrospinning combined with hot pressing to remove indigo dyes. Their results showed that the filtration efficiency for the indigo dye increased as the number of fiber membrane layers increased, and finally reached the complete removal of the dye. Wang et al. [130] electrospun a ZrO_2_ nanofiber sponge for filtering high-temperature particulate matter. At ambient temperature, the filtration efficiency of the nanosponge on aerosol particles with a diameter of 20~600 nm was 99.4%. Under the high temperature of 750 °C for PM0.3~2.5, the filtering efficiency rose to 99.97%. Moreover, they assembled a vehicle exhaust filter that could reach 98.3% particle filtration efficiency. Jiang et al. [131] electrospun a multifunctional antibacterial nanofiber membrane using soybean protein isolate (SPI)/polymide-6 (PA6)-silver nitrate solution. They proved that its filtration efficiency was more than 95% for PM0.3 particles and exhibited a good antibacterial effect, which showed its potential for high-performance air filters with antibacterial effects.

Furthermore, electrospinning materials also demonstrate unique properties and effects for sewage treatment in the environment. Apul et al. [84] electrospun a composite material composed of superfine powdered activated carbon and polystyrene, which increased the amount of adsorption of phenanthrene by 30%. Liu et al. [132] fabricated a nanocomposite membrane using silver that could filter organic dyes in water through interacting forces. Kahraman et al. [133] prepared a novel sandwich composite electrospun membrane by sandwiching a chitosan polyvinyl butyral (PVB) nanofiber between polyacrylonitrile (PAN) mats. Meanwhile, they also aminated the surface of the prepared sandwich composite and coated a layer of chitosan to form an affinity film capable of removing hexavalent chromium. The intermittent adsorption experiment on hexavalent chromium particles showed that the surface-aminated composite electrospun membrane reached the maximum adsorption capacity at pH = 2, which indicates strong promise as a wastewater treatment material. Tang et al. [134] electrospun a SiO_2_@ZrO_2_ coaxial nanofiber membrane that could be used in physical separation and electrostatic adsorption, as well as removing negatively charged particles, in wastewater. The zein nanoribbons prepared by a coaxial electrospinning device could also be used for the treatment of lead-containing wastewater [93]. The study proved that the groups on the protein molecules, containing lone pair electrons and some negative charges, adsorbed pollutants through electrostatic interaction or chelation with metal ions. Compared to those from fluid processes, the zein nanoribbons prepared using the electrospinning device had a flatter and narrower morphology, their maximum adsorption amount reached 89.37 mg/g, and they had adsorption equilibrium at 60 °C. The adsorption capacity could still be maintained at 82.3% after five cycles of reuse. For preventing drug contamination, Camire et al. [135] proposed a new method to electrospin an alkali lignin nanofiber membrane with polyvinyl alcohol, which could adsorb drug pollutants (fluoxetine) in aqueous solutions. The experiment demonstrated that lignin nanofibers with 156 nm diameter could adsorb fluoxetine in an aqueous solution in less than one hour with 70% efficiency. Another report [69] introduced a method of fabricating gelatin/PVA composite nanofiber bands loaded with bayberry tannin (GPNB-BT) to extract uranium (VI) from simulated seawater, which indicated an adsorption application prospect of electrospun nanofibers. Xiao et al. [136] obtained electrospun TiO_2_/Carbon flexible fibers that had a superior adsorption performance in removing organic dye from wastewater.

### 3.3. Recent Energy Material Applications

**Figure 5:**
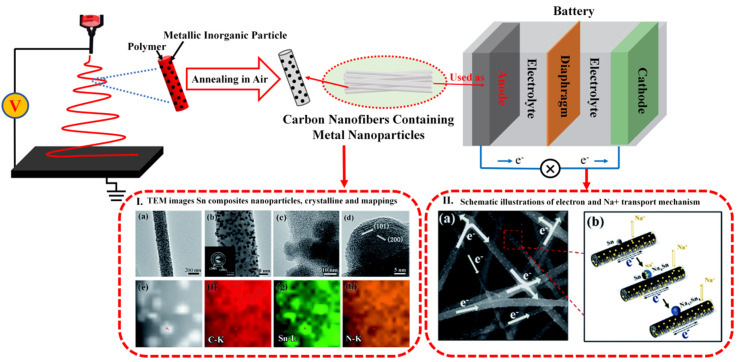
Polymer solutions can be mixed with inorganic metal nanoparticles and electrospun into ecofriendly composite nanofibers as an anode material in a battery after an annealing process. Reproduced with permission from [140]; copyright (2017) Royal Society of Chemistry.

The nanofibers of a carbon-containing composite obtained from electrospinning can be used in battery electrodes and catalytic materials due to their good conductivity, fast electron transfer, and availability of ion transport paths [89,91,137,138,139]. For example, electrospun fibers from a polymer solution mixed with inorganic metal nanoparticles can be treated through annealing for use as the positive electrode in a battery with improved long-term stable capacity (Figure 5). Sha et al. [140] obtained carbon nanofiber composites in which Sn nanoparticles were uniformly attached to the surface and loaded with nitrogen-doped carbon by using electrospinning, annealing, and reduction processes. The obtained nanofiber composite exhibited excellent electrochemical performance, with a high reversible capacity of 601 mAh/g in 200 cycles at 0.1 C, retained even after 1000 high-speed cycles, showing stable capacity when used as an anode material. Wang et al. [141] synthesized NiCoO_2_ porous nanofibers used as anode materials for lithium-ion batteries that possessed excellent storage performance, a high discharge capacity, and a high charge rate due to their special graded nanoparticle–nanofiber structure. This research team believed that their electrospun materials could not only solve the volume change problem in the lithiation/delithiation process but also provide an enlarged surface site for lithium storage and promote charge/electrolyte diffusion. Ren et al. [142] produced heat-treated SiO_2_/C fibers that had excellent conductivity and volume stability during charging and discharging due to a high specific surface area and a spatial network structure. The obtained electrospun nanofibers had a reversible capacity of 465 mAh/g at a current density of 50 mA/g in 50 cycles, much higher than pure SiO_2_, and could be used as the anode material of a lithium-ion battery. Bai et al. [143] created polyvinyl chloride nanofibers by electrospinning and carbonization to form a thousand-layer cake structure, which was beneficial to electrolyte penetration and the rapid diffusion of Na^+^. Cai et al. [144] prepared a new carboxylated multiwall carbon nanotube/carbon microbial fuel nanofiber-composite electrode using electrospinning technology. The interconnected and mechanical properties of the fibers were improved, and contact resistance was reduced by thermocompression. When this nanofiber-composite electrode was used as an anode material for microbial fuel cells, the results of the comparison to ordinary carbon nanotubes and commercial carbon felt anodes showed that the maximum power density of the nanofiber-based anodes was higher than that of ordinary carbon nanofibers and carbon felt anodes and had the highest catalytic current and exchange density, the minimum resistance, and favorable growth for rod-like bacterial cells.

A battery separator is a layer of isolation material between the positive and negative electrodes to avoid their physical contact in the battery and to prevent the battery temperature suddenly rising due to a short circuit, as well as to increase the internal pressure of the battery and prevent safety hazards such as electrolyte discharge. Therefore, the membrane must have good osmotic electrolyte properties, excellent corrosion resistance, high enough heat resistance, and mechanical strength, as well as other physical and chemical properties [145,146,147]. A sustainable polymer-fiber membrane with an excellent charge/discharge rate and high capacitance, as well as good cycle stability, has been successfully manufactured for use as a lithium-ion battery separator [147]. Electrospun polyaryl ether sulfone ketone fiber membranes possess high tensile strength and elastic modulus, good thermal stability, and high ionic conductivity (2.38 mS/cm), as well as low interfacial resistance (170 Ω). Furthermore, as a separator applied in a button cell, the membrane exhibited a high charge and discharge capacity, stable cycle performance, and high porosity [148]. In another example, the porous polyurethane/graphene oxide (PU/GO) film prepared by Liu et al. [90] showed better discharge capacity and longer cycle life than commercial separators. Lin et al. [149] also successfully fabricated polyvinylidene fluoride/poly(4-styrenesulfonic acid) lithium salt (PVDF/PSSLi) films for use as separators of lithium–sulfur batteries. It has better discharge capacity and better cycle performance than commercial separators. This is attributed to the fact that PSSLi greatly fills the gaps between the nanofibers and covers them well, creating some bonds between the molecular chains and increasing the crystallinity of the film. Thus, Li^+^ transport and sulfide ion shuttle can be promoted. Moreover, lithium-ion batteries have safety issues related to highly flammable organic electrolytes, which constrain their capability for practical applications in the next generation of high-energy batteries, although they have a strong power storage capacity. Liu et al. [11] wrapped flame retardant in a protective polymeric shell to release flame retardants when the temperature of the protective polymer case increases, which ensures the safe use of lithium-ion batteries.

### 3.4. Recent Packaging Material Applications

**Figure 6:**
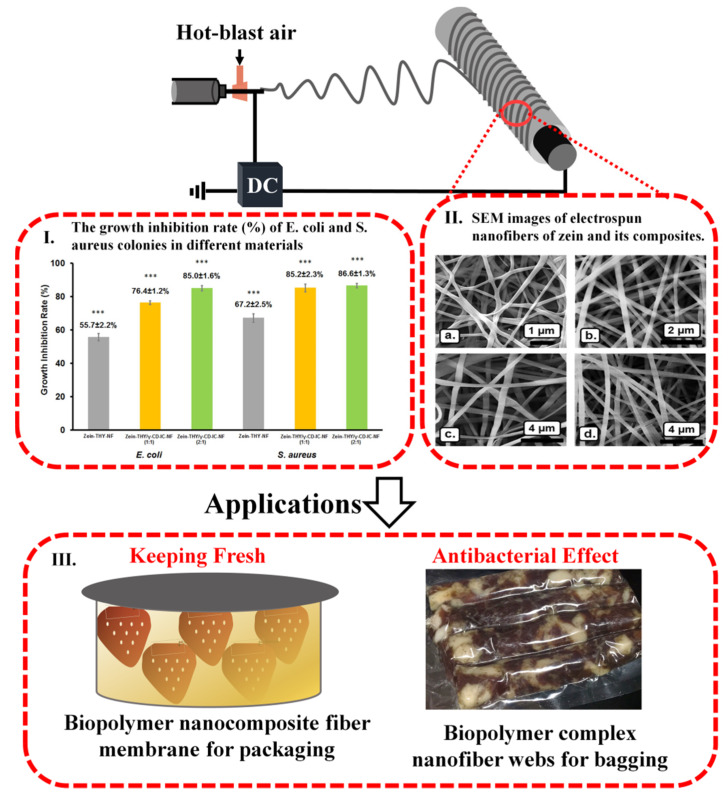
Biopolymer composite nanofibers obtained by electrospinning are used as ecofriendly packaging materials for fruits and meat, providing antibacterial properties and keeping food fresh [87]. Reproduced with permission from [87]; copyright (2017) Elsevier Ltd.

Electrospinning technology can provide new solutions for unstable bioactive ingredients in food [85,92,150,151,152]. Biopolymer composite nanofibers obtained by electrospinning can retain freshness and provide antibacterial properties when used as sustainable packaging materials for fruits and meat (Figure 6), providing the ability to better preserve food during transportation. For example, electrospun carboxymethyl chitosan/polyoxyethylene nanofiber membranes could effectively prevent strawberries from losing water, showing high gas permeability and antibacterial properties while remaining non-toxic and harmless [153]. In another study, electrospun polylactic acid/allyl isothiocyanate fiber grafted antibacterial polylactic acid film demonstrated remarkably high antibacterial activity and high mechanical properties [70]. Electrospun lentil/polyoxyethylene oxide (PEO) nanofibers with encapsulated gallic acid could enhance their oxidative stability [86]. Coating thymol/γ-cyclodextrin with an electrospun zein nanofiber mesh [87], and wrapping it in rosehip seed oil [154], as well as curcumin in zein [155] nanofibers, could all be applied in the food packaging field. Polylactic acid nanofibers encapsulated with cinnamon essential oil/β-cyclodextrin is a new type of antibacterial packaging material used in pork packaging. An electrospun zein nanofiber mesh coated with thymol [87], a nanomembrane wrapped in rosehip seed oil [154], and curcumin in zein [155], have all been used to create nanofibers with high antibacterial activity, high mechanical properties, and enhanced oxidative stability for food packaging. Both polylactic acid nanofibers encapsulated with cinnamon essential oil/β-cyclodextrin [156] and carboxymethyl chitosan/polyoxyethylene oxide nanofibers [85] were used as new types of antibacterial packaging material for pork. For the former, its minimum inhibitory concentration against *Escherichia coli* and *Staphylococcus aureus* was 1 mg/mL, and the minimum bactericidal concentration was about 7 mg/mL [156].

**Table 2:** Applications of electrospinning technology in biology, environmental protection, ecofriendly energy, and packaging materials.

Application Direction	Materials	Solvent	Operating Parameters			References
			Voltage (kV)	Distance (cm)	Flow Rate (mL/h)	
**Biology**	PLLA, Pluronic	Chloroform, DMF	18	14	0.5	[3]
	SF, PEO	HFIP	20	18	0.7–2.5	[4]
	PCL, PLGA	HFP	7.5–37.5	10–25	0.75	[5]
	PS	DMF	18	20	0.1	[7]
	PCL	DCM, TEF	15	20	0.6, 0.8, 1.6	[9]
	PEO	FA	21	10	0.8–1.2	[17]
	Gliadin, IBU	HFIP, TFA	15	-	0.2, 0.3	[18]
	Chitosan, Zein, PVP, PVA	Ethanol, Acetic acid	22	8	0.7	[19]
	KGM, PDA	Ethanol, Distillated water	16	13	0.03	[20]
	Zein, Quercetin	Ethanol	20	15	0.6	[21]
	PVP, PVB, PVPI	Ethanol	10	8	-	[22]
	PLA	HFIP	16, 12.5	23, 20	1.5, 2.5	[23,30]
	Fibrin	HFIP, Distilled water	22	10	0.5	[24]
	PCL	HFIP	16	10	2	[25]
	Poly(pro-17β- estradiol-alt-oEG)	DCM	12.5	5	0.75	[26]
	PCL, COL	HFIP	15	15	1	[27]
	PHBV, MCC	Chloroform, DMF	15	18	1	[28]
	Tecoflex EG-80A	DMF	10.5	20	0.5	[29]
	PVA, PE	Water, IPA	28	15	2	[35]
	Glycerol sebacic acid, PLLA	DCM, DMF, trichloromathane	20	-	-	[50]
	Zn(CH_3_COO)_2_·4H_2_O, Co(CH_3_COO)_2_·4H2O PVP	DMF	12	14	-	[51]
	PLG, PLA	DCM, DMF	-	-	-	[52]
	SPIR, HPMC	Ethanol, DCM	25	20	10, 30	[56]
	PCL	AA	15–38			[60]
	Al(NO_3_)_3_·9H_2_O, PVP	DI water, ethanol	15–40	30–35	15–40	[61]
	gelatin	acetic acid, distilled water, ethanol	34	-	-	[62]
	PEO, PIB, PS	Toluene, Ethanol	4–5.5	7	0.036–0.072	[63]
	PS	DMF	10–20	5–20	0.5–2	[66]
	PVB, PA6, PES	Ethanol, FA, Acetic acid	32	-	-	[94]
	PCL, Ch, Ferulic acid	DMF, THF	13	12	0.7	[80]
	COL, PVA, SA	Acetic acid	18	15	0.4	[100]
	PVP, Loratadine	Ethanol	10, 20	1, 6	5	[75]
	PU, Eudragit	DMF, THF	10, 15, 18	15	1, 1.5	[81]
	PLCL, Gelatin, NaHCO_3_	HFIP	15	23	0.8	[88]
	PCL, Gelatin	AA, FA	15	11	0.4	[106]
	PDO, PCL	HFIP	8.2–8.4	20	-	[107]
	PLCL, Gelatin	DMF, TFA	12	15	1	[109]
	ECM, PCL	HFIP	20	21	3	[111]
	PLCL, PLLA, SF	Chloroform, FA	22–23, 17	10–12, 7	0.24–0.36, 1	[112,115]
	Collagen, Ch	HFP	15	20	0.2	[110]
	SF, PLLACL	HFIP	12, 15	12, 15	0.1, 0.6	[114]
	PVA	TFA, THMs, Deionize water	10	15	-	[116]
	TSF	Deionize water	20	18	0.1–0.3	[117]
	PAN, Fe_3_O_4_ magnetic nanofiber	DMF	10, 15	10, 15	0.72, 1	[119,121]
**Environment**	PVDF, GPS	DMF	30	20	0.5	[2]
	Soy flour, PA-6	FA, Acetic acid	12–18	5–11, 20	0.2–0.3, 3	[64,129,130]
	PAN	DMF	20	10, 15, 20	0.15	[69,133]
	LPI	DMAc	7–20	12–35	0.0025–0.1	[74]
	PAN, PVP	DMAc	10, 14	15	1, 2	[77]
	PAN, PMMA	DMF	14	15	1.6	[95]
	PVDF, PTFE	DMF	30	15	0.5	[71]
	PVA, Gelatin	Ultrapure water, Glacial acetic acid	20	-	0.3	[98]
	PAN	DMF	15	10	-	[118]
	PET	TFA, DCM	5–25	8-21	1	[122]
	Nylon 6,6	DMF, FA, Chloroform	22	12	1	[126]
	PMDA, ODA	DMAc	11–14	21	0.2	[127]
	PCL, PEO	Chloroform, Acetone	25, 15	25, 15	0.3, 1	[128]
	PAN, PA-66, PES	DMF	20, 12, 75	20, 11	0.8	[79,82,83]
	PA-6	Acetic acid, FA	27–28	15	-	[131]
	SPAC, PS	DMF	40	15	1	[84]
	PVA	Deionize water	20	15	0.5	[132]
	PVA, Tetraetho ysilane, Zirconium oxychloride	DMF, Deionize water	15, 20	15, 20	0.4	[93,134]
	PVA, PVP, PAN	NaOH, Distilled water	10–25	10–20	0.6, 0.9	[135,136]
**Energy**	TPP, PVDF-HFP	DMAc, BLA	13	-	-	[11]
	CA	Acetone, DMAc	20	-	0.2	[16]
	PMMA, PAN	DMF	18	-	1.5	[138,139]
	PAN	DMF	13	16	-	[91]
	PAN	DMF	15, 25	15	0.05, 0.5, 1	[140,141]
	PVP, SiO_2_	Ethanol, DMF	16	18	1	[142]
	PAN	DMF, Acetone	18	15	1	[143]
	PVDF, PU	DMAc, EMC, Acetone, THF	30	20	0.6, 1	[145]
	PPESK	NMP, THF	13	20	-	[147]
	PVDF-HFP, PVDF	DMF, NMP	20	30	-	[148]
	PU, GO	DMF, THF	9–10	13	-	[90]
	PVDF	DMF	12	18	1	[149]
	PEO, CMCS	Distilled water	20	20	-	[153]
**Package**	PVA	Deionize water	30	12	-	[65]
	BSA, Ascorbic acid	MilliQ water	12.5	15	1	[108]
	Zein	Ethanol	11	10	0.15	[92]
	Gliadin	Acetic acid	18	10	1	[152]
	Zein	Ethanol	15	10	1	[85]
	PEO, Lentil powder	Chloroform, Ethanol	20	8	0.5	[70]
	PEO,	Chloroform	15	30	0.6	[86]
	Zein, Thymol	DMF	17	17	0.5	[87]
	Zein, Gelatin	Acetic acid, Ethanol	14–16	12	0.1, 0.3–0.7	[154,155]
	PLA, CEO, Ch, Glucose oxidase	DCM, DMF	12–16	10–14	2–2.4	[156]

Abbreviations: DMF, *N*,*N*-dimethylformamide; IBU, ibuprofen or 2-(4-isobutylphenyl)propanoic acid; HFIP, hexafluoroisopropanol; TFA, trifluoroacetic acid; KGM, konjac glucomannan; BLE, acetone; PDA, poly dopamine; PVPI, poly(vinyl pyrrolidone)-iodine; PLA, polylactide; IPA, isopropanol; PHBV, poly(3-hydroxybutyrate-co-3-hydroxyvalerate); MCC, microcrystalline cellulose; SPIR, spironolactone; HPMC, hydroxypropylmethylcellulose; DCM, dichloromethane; PIB, polyisobutylene; PS, polystyrene; Ch, chitosan; THF, tetrahydrofuran; COL, collagen; SA, salicylic acid; TFA, trifluoroacetic acid; PA6, polyamide-6; PES, polyester; ECM, extracellular matrix; HFP, 1,1,1,3,3,3-hexafluoro-2-propanol; PLLACL, poly (l-lactide-co-caprolactone); THMs, trichloromethane; TSF, tussah silk fibroin; PMMA, polymethylmethacrylate; PPESK, poly(phthalazinone ether sulfone ketone); PDO, polydioxanone; NMP, N-methyl pyrrolidone; CA, cellulose acetate; PVDF, polyvinylidene fluoride; PTFE, polytetrafluoroethylene; PET, polyethylene terephthalate; PMDA, pyromellitic dianhydride; ODA, 4, 4′-oxydianiline; DMAc, *N*,*N*-dimethylacetamide; SPAC, superfine powdered activated carbon; PU, polyurethane; CEO, cinnamon essential oil; DCM, dichloromethane; AA, glacial acetic acid; BSA, bovine serum albumin.

## 4. Conclusions

In this review, we introduced current important electrospinning devices and expounded upon the various eco-friendly and sustainable composite nanofibers and membranes that can be obtained from their setups, as well as the applications of these nanomaterials. The factors influencing the quality and features of these materials were also discussed. We recognize that electrospinning technology still has problems to be solved [157]. Among these, the toxicity of most of the solvents used requires us to find environmentally friendly substitutes. In addition, electrospinning equipment needs to be improved to be more suitable for large-scale industrial applications. We envisage that with many new electrospinning techniques, optimizing and upgrading more novel, finer, and uniform nanofiber composites could happen in the near future, which will be not only used in biomedical sciences and engineering but also broadly useful in various sustainable fields. For example, recent reports have shown that electrospinning technology could provide novel solutions to the unstable bioactive components in foods to keep food better preserved during transportation. All the new developments implicate that electrospinning technology will play an important role in future sustainable science and engineering technology.
